# Carbapenem-resistant *Klebsiella pneumoniae* in high-risk haematological patients: factors favouring spread, risk factors and outcome of carbapenem-resistant *Klebsiella pneumoniae* bacteremias

**DOI:** 10.1186/s12879-017-2297-9

**Published:** 2017-03-10

**Authors:** Alessandra Micozzi, Giuseppe Gentile, Clara Minotti, Claudio Cartoni, Saveria Capria, Daniele Ballarò, Stefania Santilli, Emanuele Pacetti, Sara Grammatico, Giampaolo Bucaneve, Robin Foà

**Affiliations:** 1 Dipartimento di Biotecnologie Cellulari ed Ematologia, Sapienza Università di Roma, Via Benevento 6, 00161 Rome, Italy; 2 Azienda Policlinico Umberto I, Rome, Italy; 3 Ospedale S. Maria della Misericordia, Perugia, Italy

**Keywords:** Carbapenem-resistant *Klebsiella pneumoniae*, Haematological malignancies, Neutropenia, Spread, Bacteremia

## Abstract

**Background:**

Carbapenem-resistant *Klebsiella pneumoniae* (CRKP) spread and infections in patients with haematological malignancies are a serious concern especially in endemic areas. Treatment failures and delay in appropriate therapy for CRKP infections are frequent and the mortality rate associated with CRKP bacteremia in neutropenic haematological patients is reported about 60%.

**Methods:**

Haematological patients harboring CRKP hospitalized between February 2012 and May 2013 in an Italian Teaching hospital were examined. Conditions favouring CRKP spread in a haematological unit, risk factors for bacteremia in CRKP-carriers and for CRKP bacteremia-related death were evaluated in this observational retrospective study.

**Results:**

CRKP was isolated in 22 patients, 14 (64%) had bacteremia. Control measures implementation, particularly the weekly rectal screening for CRKP performed in all hospitalized patients and contact precautions for CRKP-carriers and newly admitted patients until proved CRKP-negative, reduced significantly the CRKP spread (14 new carriers identified of 131 screened patients vs 5 of 242 after the intervention, *p* = 0.001). Fifty-eight percent of carriers developed CRKP bacteremia, and acute myeloid leukemia (AML) resulted independently associated with the bacteremia occurrence (*p* = 0.02). CRKP bacteremias developed mainly during neutropenia (86%) and in CRKP-carriers (79%). CRKP bacteremias were breakthrough in 10 cases (71%). Ten of 14 patient with CRKP bacteremias died (71%) and all had AML. The 70% of fatal bacteremias occurred in patients not yet recognized as CRKP-carriers and 80% were breakthrough. Initial adequate antibiotic therapy resulted the only independent factor able to protect against death (*p* = 0.02).

**Conclusions:**

The identification of CRKP-carriers is confirmed critical to prevent CRKP spread. AML patients colonized by CRKP resulted at high risk of CRKP-bacteremia and poor outcome and the adequacy of the initial antibiotic therapy may be effective to improve survival. To limit the increase of resistance, the extensive use of antibiotics active against CRKP should be avoided, but in the setting of high CRKP pressure and high-risk CRKP-colonized haematological patients, timely empiric antibiotic combinations active against CRKP could be suggested as treatment of febrile neutropenia.

## Background

The diffusion of carbapenem-resistant *Klebsiella pneumoniae* (CRKP) [1–5] in the setting of patients with haematological malignancies undergoing intensive myelosuppressive chemotherapy is a very worrying challenge [6–11], especially in endemic areas. The most important mechanism in carbapenem resistance in Enterobacteriaceae is the production of carbapenemases, primarily KPC VIM NDM and OXA-48 types, which display remarkable geographycal variability. KPC production is the most frequent mechanism of resistance to carbapenems in *K. pneumoniae* in Italy, as already reported in the 2011 Italian survey [4] and KPC-type carbapenemases-producing *K.* *pneumoniae* has become endemic, as very recently confirmed [5]. Underlying haematological disease, intensive chemotherapy, neutropenia, gastrointestinal mucositis and prolonged hospitalization are all conditions favouring the CRKP spread and infections, mainly bacteremias [6–11]. Treatment failures and delay in appropriate therapy for CRKP infections are predictable since the recommended empiric antibiotic treatments for febrile neutropenia, both monotherapy and combinations [12–14], in most cases contain antibiotics without in vitro activity against CRKP. The mortality rate associated with CRKP bacteremia in neutropenic haematological patients is reported about 60% [6–11], and expected to remain very high, especially in particularly vulnerable patients such as those with acute leukemia. Notwithstanding, clinical data about the impact of CRKP in this high-risk population are still scant in literature. In this observational study on patients with haematological malignancies conducted over a 17-month period, we aimed to identify conditions favouring CRKP spread in a haematological unit, assess risk factors for bacteremia in haematological patients colonized with CRKP, and analyse risk factors for poor outcome among haematological patients with CRKP bacteremia. We also evaluated the impact of infection control program implementation on new CRKP acquisitions in our Haematology Department.

## Methods

All patients with haematological malignancies infected or colonized with CRKP, hospitalized between 24 February 2012 and 31 May 2013 at the Hematology Department of the *Sapienza* University of Rome, were included in the study.

### Setting

The Hematology Department, located in a four-storey building, consists in 1 Pediatric and 2 Adult Wards (Unit A and Unit B) each with 14 beds in double-occupancy rooms, 1 Transplant Unit (5 single bedrooms), 1 Short Hospitalization Unit (5 beds), Emergency Rooms and Day Hospital. On July and August 2012, Unit A closed for renovations.

During the study period, each ward had its own medical, nursing and ancillary staff who took care of both CRKP-positive and negative patients, while during the night shift the medical staff was the same for the entire Hematology Department.

### CRKP spread, surveillance and isolation precautions

For all CRKP-positive patients included in the study, the following informations about hospitalization periods, before and after the documentation of CRKP acquisition, were collected: a) the presence and the number of CRKP-positive patients hospitalized in the same time; b) the occurrence of CRKP bacteremia in other patients; c) the occurrence of the death of a CRKP-positive patient; d) the transfer from or to other Hospitals or Departments. Patients with a positive rectal swab screening, in the absence of any sign or symptom of infection, were defined as colonized by CRKP. CRKP horizontal transmission during the current hospitalization was hypothesized for those CRKP-positive patients who had a negative screening at admission, or had not been transferred from or to other Hospitals or Departments. Before March 2012, surveillance cultures from rectum, pharynx, genitourinary tract and nasal cavity to detect multidrug-resistant (MDR) bacteria and fungal colonization were usually collected in neutropenic hospitalized patients. From March 2012, infection control measures were implemented in Unit B: the screening for CRKP rectal colonization [15, 16] was performed weekly in all hospitalized patients and CRKP-positive patients were kept under contact precautions [17] in isolation rooms or cohorted in double-occupancy rooms. From September 2012, the rectal screening for CRKP carriage detection was extended to all patients hospitalized in the Hematology Department, weekly during hospitalization, prior to admission and at entry. Newly admitted patients not screened before hospitalization were placed under contact precautions until the results of surveillance cultures performed at entry were available and proved negative. All healthcare workers received a specific training on the relevance of CRKP and its routes of transmission.

### Patients and antibacterial therapy

Medical records of all patients harboring CRKP were reviewed. Gender, age, underlying haematological malignancy, chemotherapy, number of hospitalizations, length of hospitalization and neutropenia prior to CRKP acquisition, duration of CRKP-positive patient status (colonized or infected) during the study period (from the first positive microbiological result, during hospitalizations and out-patient condition, until the end of the study period or the discontinuation of any chemotherapeutic course and/or hospitalization) and previous hospitalizations, were recorded in all CRKP-positive patients. In patients with CRKP bacteremia, length of CRKP colonization and neutropenia before bacteremia, neutrophil count at the onset of bacteremia, antibacterial treatments (initial therapy and subsequent modifications) and outcome were also recorded.

CRKP bacteremia was defined breakthrough when blood-coltures yelding CRKP were taken in a patient already receiving an intravenous antibacterial therapy initiated by at least 24 h.

An antibacterial regimen was considered adequate when it included at least 1drug displaying in vitro activity against the CRKP isolate.

CRKP colonized patients who developed bacteremia and those who did not, and patients with CRKP bacteremia who died and those who did not were compared.

### Microbiological studies

The CRKP isolates studied were at least one per patient (either the first colonizing or/and the first blood isolate identified). Species identification and MIC determination were performed using BD Phoenix automated microbiology system (Becton Dickinson Italia S.p.A Milano, Italy). MICs of imipenem, meropenem, ertapenem, gentamicin, colistin and tigecycline were also evaluated using Etest (BioMerieux Italia S.p.A., Firenze Italy) in accordance with the manufacturer’s instructions. Results are interpreted in accordance with the last breakpoints proposed by the European Committee on Antimicrobial Susceptibility Testing (EUCAST) [18].

### Statistical analysis

Continuous variables were compared using Kruskal-Wallis test. Categorical variables were compared with Fisher’s exact test; odds ratios (ORs) and 95% confidence intervals (CIs) were calculated.

Forward stepwise logistic regression models were used to assess the relative importance of the various prognostic factors that could influence the occurrence of bacteremia in CRKP colonized patients (acute leukemia and acute myeloid leukemia [AML], intensive chemotherapy, female gender, >/=7 days spent colonized by CRKP with <100 neuthrophils/mmc) and the occurrence of death in patients with CRKP bacteremia (underlying disease, intensive chemotherapy, initial adequate therapy, neutrophils recovery >1000/mmc within 72 h after bacteremia onset, patient identified as CRKP carrier at bacteremia onset, occurrence of breakthrough bacteremia within 48 h of ongoing antibiotics).

To identify risk factors for mortality, a multivariate analysis using Cox forward regression and calculation of Hazard Ratios with 95% CIs was also performed. Survival curves were constructed using the Kaplan-Meier method and were compared using the log-rank test.

## Results

From 24 February 2012 to 31 May 2013, a CRKP was isolated in 22 patients, 14 (64%) developed a CRKP bacteremia and 8 (36%) remained as CRKP rectal carriers.

**Fig. 1:**
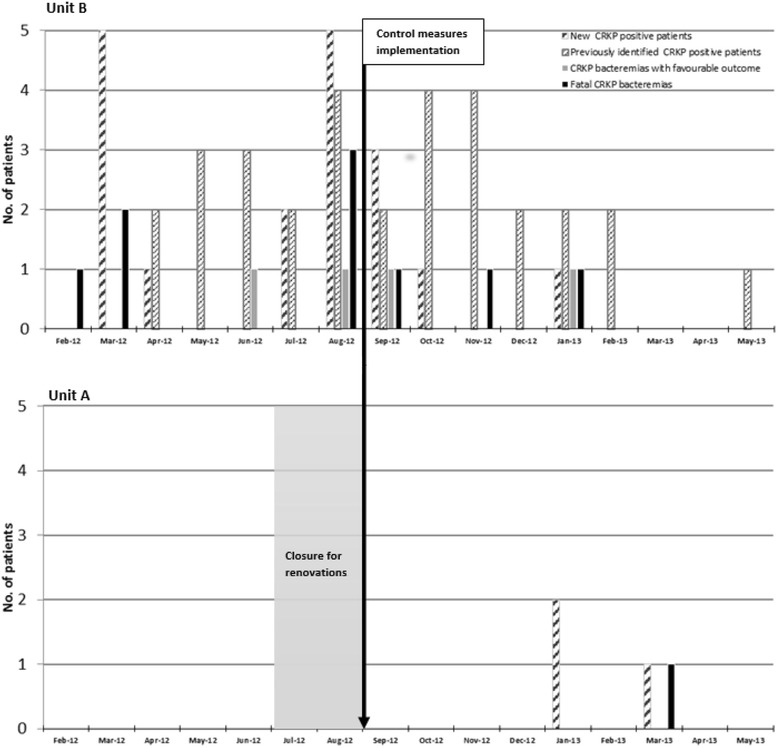
Spread of CRKP in the Hematology Department during the study period (February 2012 - May 2013)

The spread of CRKP in the Hematology Department is chronologically described in Fig. 1. The first 18 patients who acquired CRKP (colonized or infected) were hospitalized in Unit B, the last 3 patients in Unit A.

The first CRKP isolate was detected on 24 February 2012 at Unit B, in the blood of an AML patient who died from CRKP bacteremia before blood-culture results were available (during a previous hospitalization on January 2012, the patient had been treated for a severe ESBL-producer *E.coli* cellulitis with prolonged meropenem therapy and surgical dressings performed in another Department). All the patients who were hospitalized in Unit B were screened for the control of CRKP spread and 5 patients were identified as CRKP rectal carrier (March 2012). In particular, while the second CRKP rectal carrier identified was hospitalized after the death of the first CRKP-positive patient (the positive swab was collected 1 week after entry), the third CRKP rectal carrier identified had been transferred from another Department on 7 February 2012, therefore the patient was hospitalized prior to and together with the two previously described positive patients.

Regarding Unit A, the first CRKP-positive patient was identified on January 2013. The patient, transferred from another Department, resulted CRKP rectal carrier at entry and died 10 days after from the underlying haematological disease. The second CRKP rectal carrier identified, in the period preceding the documentation of the CRKP colonization, was either hospitalized in the same time of the first one either submitted to an invasive procedure in another Department.

### Surveillance of CRKP spread

From March to August 2012 (6-month period), 341 rectal swabs for CRKP screening were collected from 131 hospitalized patients (90 hospitalized in Unit B) (mean: 57 swabs per month) and 14 new carriers were identified (2.3 per month, 11% of all screened patients, 16% of patients hospitalized in Unit B). During the subsequent 9-month period (September 2012-May 2013) after the implementation of the infection control program, 1398 rectal swabs, 104 collected prior to hospitalization, (mean:155 swabs per month) identified 5 new CRKP carriers among 242 patients screened (0.6 per month, 2% of all screened patients) (14 new CRKP carriers of 131 screened patients vs 5 of 242, *p* = 0.001).

The susceptibilities to antibiotics of the 22 *K.* *pneumoniae* isolates are shown in Table 1.

**Table 1:** Susceptibilities to antibiotics of the 22 *K.* *pneumoniae* isolates

Antimicrobial agent	MIC range μg/mL	MIC _50_ μg/mL	MIC _90_ μg/mL	N.(%) of isolates that were susceptible
imipenem	>32	>32	>32	0
meropenem	>32	>32	>32	0
ertapenem	>32	>32	>32	0
colistin	0.125 to 32	0.75	24	12 (54.5)
tigecycline	0.5 to 8	2	4	6 (27.2)
gentamicin	3 to >256	8	>256	0

### Characteristics of patients harboring a CRKP and possible route of CRKP acquisition

The characteristics of the 22 CRKP-positive patients are described in Table 2. CRKP was first isolated as a rectal colonizer without any infection sign or symptom in 19 (86%) patients, while in 3 (14%) CRKP was first isolated in the blood.

**Table 2:** Characteristics of the 22 patients harbouring carbapenem-resistant *K.* *pneumoniae*

	N°	%
PATIENTS	22	
Male gender	13	59
Age (years), mean (range)	53.7 (28–76)	
N° of patients who developed CRKP bacteremia	14	64
- Previously identified as CRKP rectal carriers	11	
N° of patients who remained CRKP rectal carriers	8	36
UNDERLYING HAEMATOLOGIC MALIGNANCY		
Acute leukemia	16	73
- Acute myeloid leukemia	12	55
Other haematologic malignancy	6	27
CHEMOTHERAPEUTIC TREATMENT	20	91
Intensive remission induction/reinduction	8	40
Intensive remission consolidation	4	20
Other chemotherapy	8	40
CHARACTERISTICS OF PATIENTS AT CRKP ACQUISITION (n° of patients)		
Rectum as first site of CRKP isolation	19	86
Blood as first site of CRKP isolation	3	14
Ongoing fluoroquinolones oral prophylaxis	11	50
Ongoing systemic antibacterial treatment (carbapenem)	9 (1)	41
Carbapenems within the last 4 weeks	5	23
<1000 neutrophils/mm3	12	55
<100 neutrophils /mm3	10	45
days with < 1000 neutrophils/mm^3^ before CRKP acquisition: mean, median (range),	19.2,13 (2–53)	
days with < 100 neutrophils /mm^3^ before CRKP acquisition: mean, median (range)	10.2, 4.5, (1-53)	
N° of patients negative at CRKP rectal screening performed at entry:	19	86
First hospitalization (n° of patients)	14	64
Transferred from other Departments	2	
Prior hospitalization (n° of patients)	5	23
During the period preceding CRKP acquisition:		
Mean, median (range) days of previous hospitalization	28, 17 (7–60)	
n°of patients hospitalized together with other CRKP-positive patients	17/19	
n°of patients hospitalized together with 4 or more CRKP carriers	11/19	86
n°of patients hospitalized together with 9 or more CRKP carriers	6/19	64
n°of patients hospitalized when clinical emergencies occurred in other CRKP-positive patients ^a^	14/19	
CRKP bacteremia occurrence	14/19	
death for any cause in at least 1 CRKP-positive patients	10/19	
n° of patients temporary transferred to other Departments for invasive ﻿procedures﻿	4/19	
N° of patients positive at CRKP rectal screening performed at entry	3	14
Previous hospitalization	3	
Transferred from other Departments (n° of patients)	2	
In the same Unit	1	

^a^5 patients had the first CRKP-positive rectal sample collected the same day in which a CRKP bacteremia and/or the death of a CRKP-positive patient occurred in the ward were they were hospitalized

Seventeen patients (77%) experienced only one hospitalization as CRKP positive patients: 12 patients died during the hospitalization (10 AML patients for CRKP bacteremia and 2 patients for causes not related to CRKP) and 5 completed the chemotherapy program and in the subsequent study period, they have not been hospitalized. Five patients (23%) experienced more hospitalizations as CRKP-positive patient (mean 5, median 4, range 3–8, hospitalizations per patient).

Overall, in the 22 patients the median duration of CRKP-positive patient condition (colonized or infected) was 12 days (mean 70.7 days, range 2–440 days). The 10 AML patients who developed a fatal CRKP bacteremia, spent a mean of 11.6 days (median 5, range 3–48 days) as CRKP-positive patient. Seven patients who have concluded the chemotherapy program before the end of the study spent a mean of 50.1 days (median 24, range 4–162) as CRKP-positive patient, while 3 patients remained CRKP carriers until the end of the study survey (mean 358 days, median 322, range 314–440).

Nineteen patients (86%) were negative at hospital admission (14 were hospitalized for the first time; 5 had been previously hospitalized, 4 in the same Unit) and data regarding the hospitalization period preceding the CRKP acquisition (colonization or bacteremia) are shown in Table 2. Seventeen (89%) patients were hospitalized at the same time of other CRKP-positive patients (11 together with 4 or more CRKP-positive patients), 14 (74%) when a CRKP bacteremia developed in other patients, 10 (53%) when one or more CRKP-positive patient died (notably 8 patients resulted new CRKP carriers within 3 days following bacteremia or death occurrence). As additional potential risk factor for CRKP acquisition, 4 of the 19 patients who were negative at admission, had been temporarily transferred to other Departments for invasive procedures (Table 2).

Only 3 of the 22 CRKP-positive patients (14%) resulted CRKP carriers at entry, and all of them had been previously hospitalized. Overall, the possible acquisition of a CRKP outside the Hematology Department (previous hospitalization, transfer from other Departments, temporary transfer to other Departments) could be assumed in 7 out of 22 patients (32%) (Table 2).

### Characteristics and risk factors for CRKP bacteremia in colonized patients

Eleven out of 19 (58%) CRKP rectal carriers developed CRKP bacteremia after a median of 6 days (mean 38.5 days, range 1–180) from the first CRKP-positive rectal swab. Among these, 5 patients who had previously been screened negative, resulted CRKP carriers only after the onset of chemotherapy-related neutropenia and fluoroquinolone prophylaxis initiation, and they developed the CRKP bacteremia within 48 h from the collection of the first CRKP-positive swab.

CRKP colonized patients who developed bacteremia, and those who did not are compared in Table 3. At the forward stepwise logistic regression model, only AML status resulted an independent risk factor for the occurrence of bacteremia in colonized patients (Table 4).

**Table 3:** Risk factors for bacteremia in the 19 patients colonized with carbapenem-resistant *K.* *pneumoniae* (CRKP) at univariate analysis

	Bacteremia	No bacteremia	*p*
N° of CRKP colonized patients	11	8	
Male	5 (45%)	8 (100%)	
Female	6 (54%)	0	0.01
Underlying disease			
Acute leukemia	10 (91%)	3 (37.5%)	0.0075
Acute myeloid leukemia	9 (82%)	0	0.0007
Other haematological malignancy	1 (9%)	5 (62.5%)	ns
Chemotherapy	7 (64%)	5 (62.5%)	ns
- Intensive induction chemotherapy	6 (54%)	0	0.01
n° of patients with <1000 neutrophils/mm3 at CRKP acquisition	5 (45%)	4 (50%)	ns
n° of patients with <100 neutrophils/mm3 at CRKP acquisition	4 (36%)	3 (37%)	ns
Days spent colonized by CRKP with:			
< 1000 neutrophils/mm3, mean, median (range)	14.8, 10 (0–78)	10.7, 5.5 (0–30)	<0.01
< 100 neutrophils/mm3, mean, median (range)	9.1, 8 (0–30)	5.6, 3 (0–15)	<0.01
Rectal colonization	11 (100%)	8 (100%)	ns
Single site colonization	4 (36%)	5 (62.5%)	ns
Multiple sites colonization	7 (64%)	3 (37%)	ns
Colonized sites besides the rectum			
vagina/urethra	3	0	
urinary tract	5	2	
oral cavity, pharynx	2	2	
Mucositis	6 (54%)	3 (37%)	ns
- Diarrea	6	3	
CRKP localized infection	7 (64%)	3 (37.5%)	ns
vaginitis	3	0	ns
typhlitis	2	1	
hemorroidal cellulitis	2	1	
cellulitis	0	1	
Previous febrile neutropenia during CRKP colonization	6	3	ns
Previous bacteremia other than CRKP bacteremia during CRKP colonization	4	2	ns
Previous broad-spectrum antibiotics during CRKP colonization	5	4	ns

**Table 4:**
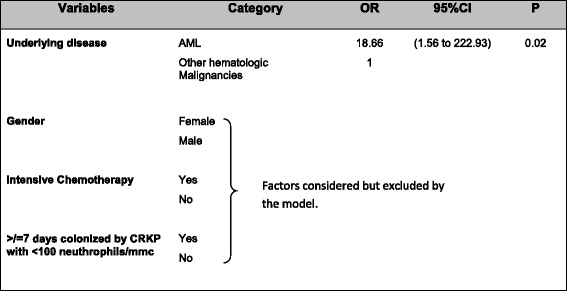
Forward stepwise logistic regression model of risk factors for bacteremia in carbapenem-resistant *K.* *pneumoniae* (CRKP) colonized patients

### CRKP bacteremias and risk factors for death

During the 17-month study period, CRKP caused 14 bacteremias, which represented the 12.5% of all Gram-negative bacteremias diagnosed in patients attending the Hematology Department. In particular, the incidence of CRKP among all gram-negative bacteremia documented in AML patients was 30% (9/30) compared with 5% (5/96) in the other haematological patients (*p* = 0.0007).

The characteristics of patients with CRKP bacteremia are shown in Table 5. The majority of patients had acute leukemia (93%), mostly AML (86%), were neutropenic (86%) and were CRKP rectal carriers (79%). Eleven patients (79%) developed CRKP bacteremia during the first hospitalization as CRKP-positive patient. CRKP bacteremia was breakthrough in 10 cases (71%) developing in patients already receiving an empiric intravenous treatment for febrile neutropenia, initiated by 48 h or less in 7 cases (43%). The ongoing antibiotics were piperacillin-tazobactam in 5 cases, piperacillin-tazobactam plus tigecycline in 2, meropenem in 2, ceftriaxone in 1.

**Table 5:** Carbapenem-resistant *K.* *pneumoniae* bacteremias

	N°	%
N° of cases	14	
Mean age, years (range)	51.5 (28–68)	
Male/female	5/9	
Acute leukemia	13	93
Acute myeloid leukemia	12	86
Other haematologic malignancy	1	7
Intensive Induction /reinduction chemotherapy	6	54
Intensive consolidation chemotherapy	4	29
Days of hospitalization before bacteremia, mean, median (range),	22.8, 17 (1–90)	
No CRKP rectal carriers (n° of patients)	3	21
CRKP rectal carriers (n° of patients)	11	79
CRKP carrier from ≤ 2 days (n° of patients)	5	36
Days spent CRKP colonized before bacteremia, mean, median, (range)	38.5, 6, (1–180)	
Multiple-sites CRKP colonization	6	54
CRKP colonization acquired in the current hospitalization	8	57
CRKP colonization acquired during a previous hospitalization	3	21
Documented source of bacteremia	7	50
labia and vaginal cellulitis	3	21
hemorroidal cellulitis	1	7
typhlitis	2	14
lung	1	7
Bacteremia of unknown origin (CRKP carriers)	7 (4)	50
Previous antibiotic treatment	13	93
Piperacillin-tazobactam monotherapy	11	79
Tigecyclin containing combination	8	57
Carbapenem containing combination	3	21
N° of patients with <1000 neutrophils/mmc at the onset of bacteremia	12	86
N° of patients with < 100 neutrophils/mmc at the onset of bacteremia	11	79
Shock	8	57
Persistent bacteremia (≥3 days)	9	64
- days of bacteremia, mean (range)	4.6 (3–10)	
Breakthrough bacteremia	10	71
- days of antibiotic treatment before CRKP bacteremia onset: mean, median (range)	3.6, 2 (1–10)	
Overall (initial or subsequently modified) adequate^a^ antibiotic treatment	9	64
Initial adequate antibiotic treatment	6^b^	43
One “in vitro” active antibiotic	5^c^	36
More than 1 in vitro active antibiotics	1^d^	7
Response to adequate initial treatment	2	14
Initial inadequate antibiotic treatment	8^e^	57
Modification of inadequate initial treatment (within 48 h)	7 (6)	50
Modification with adequate antibiotics	3	21
Response to antibiotic treatment modification	0	
Deaths	10	71
Mean days for death (range)	4.6 (2–12)	-
Early death (within 72–96 h)	5	36
Shock	7	50
Breakthrough bacteremia	8	57

^a^The antibacterial regimen included at least 1 drug displaying in vitro activity against the CRKP isolate

^b^The CRKP bacteremia was breakthrough in 3 cases

^c^colistin in 1 case, tigecyclin in 4 cases

^d^colistin + tigecycline

^e^The CRKP bacteremia was breakthrough in 7 cases

Overall, 64% (9 of 14) of patients with CRKP bacteremia received as initial or subsequently modified treatment, at least 1 drug in vitro active against the CRKP isolate (Table 5).

At the onset of CRKP bacteremia, 8 patients (57%) did not receive any antibiotic in vitro active against CRKP while 6 patients (43%) were initially treated with at least 1 drug active against CRKP. Of these, 3 were recognized as CRKP-carriers and were receiving empiric antibiotic treatment, the sudden onset of shock and high fever led to the rapid change of the ongoing treatment according to the in vitro susceptibility of the colonizing CRKP isolate and blood cultures proved CRKP breakthrough bacteremia occurrence in all the cases.

Overall, 12 of the 22 (55%) patients infected or colonized by CRKP died: 10 of CRKP bacteremia (45%), 1 of MDR *Acinetobacter baumannii* bacteremia and 1 of the underlying haematological malignancy.

CRKP bacteremias were fatal in 71.4% of cases (10/14), all fatal bacteremia occurred in AML patients and during the first hospitalization as CRKP-positive patient, and the 50% of patients died within 72–96 h from bacteremia onset (Table 5). Patients with CRKP bacteremia who died and those who survived are compared in Table 6. In particular, the rates of patients colonized, and of those colonized at single (rectum) or multiple body sites, were similar in survivors and non-survivors. However, all the 7 patients who had not been identified as CRKP carriers at the onset of bacteremia (1 not screened, 2 true negative and 4 without the results of the first CRKP positive rectal screening available yet) died for bacteremia, and 6 had received an inadequate initial treatment.

**Table 6:** Risk factors for death in patients with carbapenem-resistant *K.* *pneumoniae* bacteremia at univariate analysis

	survivors	nonsurvivors	*p*
N° of patients	4 (29%)	10 (71%)	
Age, years; mean, median (range)	46, 48 (28–59)	54, 54 (35–68)	0.001
male	2	3	ns
female	2	7	
Acute myeloid leukemia	2 (50%)	10 (100%)	0.06
Induction/reinduction intensive chemotherapy	0	6 (60%)	0.08
Steroids	4 (100%)	10 (100%)	ns
Neutrophils count at the onset of bacteremia (n° of patients)			
< 1000 neutrophils/mmc	3 (75%)	9 (90%)	ns
< 100 neutrophils /mmc	3 (75%)	8 (80%)	ns
days of neutropenia before bacteremia, mean, median (range)			
with <1000 neutrophils/mmc	2.7, 2 (0–7)	10.4, 7 (0–35)	0.002
with <100 neutrophils/mmc	2.7, 2 (0–7)	4.8, 4 (0–21)	0.01
Neutrophil recovery (>1000/mmc) within 72 h from the onset of bacteremia (n° of pts)	3 (75%)	0	0.004
CRKP rectal carrier	4 (100%)	7 (70%)	ns
One-site colonization	2 (50%)	4 (40%)	ns
Multiple-sites colonization	2 (50%)	3 (30%)	ns
No CRKP rectal carrier	0	3 (30%)	ns
Patients not identified as CRKP carriers at the onset of bacteremia	0	7^a^ (70%)	0.06
Documented source of bacteremia	2 (50%)	5 (50%)	ns
Fever of unknown origin	2 (50%)	5 (50%)	ns
CRKP carrier	2 (50%)	2 (20%)	ns
Breakthrough bacteremia	2 (50%)	8 (80%)	ns
occurrence within 48 h of ongoing antibiotics	0	6 (60%)	0.08
Persistent bacteremia (≥3 days)	2 (50%)	7 (70%)	ns
days of bacteremia, mean, median (range)	2.5, 2 (1–5)	3.7, 3.5 (1–10)	0.003
Shock	2 (50%)	6 (60%)	ns
Overall adequate antibiotic therapy^b^	4 (100%)	5 (50%)	ns
Initial adequate antibiotic therapy^b^	4^c^ (100%)	2^d^ (20%)	0.001
Modification of the initial antibiotic therapy	2^e^ (20%)	7^f^ (70%)	ns
- with adequate antibiotic therapy^b^	2	3	ns
in vitro colistin-resistant CRKP isolate	1	6	ns
in vitro colistin - and tigecycline resistant CRKP isolate	0	5	ns
in vitro colistin-, tigecyclin- and gentamycin-resistant CRKP isolate	1	5	ns

^a^1 patient was not screened, 2 resulted negative, in 4 patients the first positive result was not available

^b^The antibacterial regimen included at least 1 drug displaying in vitro activity against the CRKP isolate

^c^tigecyclin in 3 cases, colistin in 1 case

^d^tigecyclin and tigecyclin + colistin combination in 1 case each

^e^colistin and colistin + gentamicin combination in 1 case each

^f^tigecycline in 2 cases, tigecycline + meropenem combination in 2 cases, colistin + gentamicin + tigecycline combination in 3 cases

**Fig. 2:**
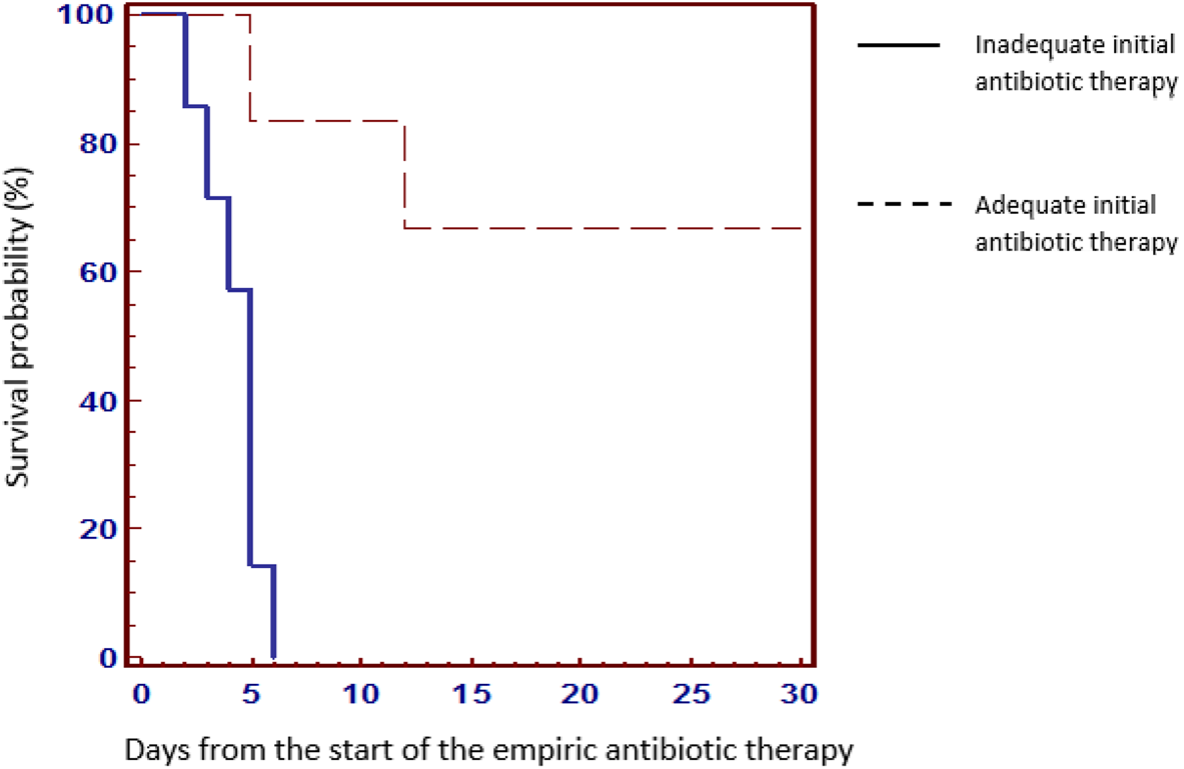
Kaplan–Meier curves showing the impact of initial adequate antibiotic therapy on survival at 30 days in patients with CRKP bacteremia (log rank test =9.17, *p* = 0.002)

Mainly because of the limited sample size, no risk factors for mortality could be included in the forward stepwise logistic regression model. In the Cox regression calculation (Table 7) only the initial adequate antibiotic therapy resulted an independent factor able to protect against 30-days mortality. Kaplan-Meier curves confirmed that patients treated with initial adequate antibiotic therapy had higher 30 days survival rates, if compared to those initially treated with inadequate therapy (log rank test =9.17; *p* = 0.002) (Fig. 2).

**Table 7:** Multivariate models of risk factors for 30-day crude mortality in patients with carbapenem-resistant *K.pneumoniae* bacteremia

	HR (95% CI)	*p*
Model1		
• Initial adequate therapy	0.08 (0.01 to 0.72)	0.02
• Pt not identified as CRKP carrier at onset	*Not included in the model*	
• Neutrophil recovery (>1000/mmc) within 72 h from the onset of bacteremia	*Not included in the model*	
Model2		
• Initial adequate therapy	0.08 (0.01 to 0.72)	0.02
• Breakthrough bacteremia occurrence within 48 h of ongoing antibiotics	0.17 (0.01 to 2.58)	0.20
• Intensive Chemotherapy	10.9 (0.73 to 162.8)	0.08
• AML	*Not included in the model*	
Model3		
• Initial adequate therapy	0.05 (0.002 to 0.86)	0.04
• Breakthrough bacteremia occurrence within 48 h of ongoing antibiotics	0.17 (0.01 to 2.58)	0.20
• Intensive Chemotherapy	10.9 (0.73 to 162.8)	0.08
• Pt not identified as CRKP carrier at onset	*Not included in the model*	
• Neutrophil recovery (>1000/mmc) within 72 h from the onset of bacteremia	*Not included in the model*	
• AML	*Not included in the model*

## Discussion

The implementation of control measures to limit the spread of CRKP, particularly the careful weekly screening of CRKP rectal colonization aimed to the prompt identification of CRKP positive patients, together with the isolation procedures extended to newly admitted patients still not screened, proved effective. After the intervention, the rate of new CRKP-positive among hospitalized patients decreased from 11 to 2%, from 2.3 to 0.8 new CRKP-positive patient per month. The detection of the 45% of CRKP-positive patients during the 3-month period of bed shortage, when the urgency of hospitalization might have reduced the accuracy of isolation procedures - particularly for not screened newly admitted patients - corroborates this result.

Even if not confirmed by genotyping analysis of the CRKP isolates, it is possible that the failure to recognize the first CRKP carrier might have been responsible of the CRKP spread in the Hematology Department, stressing further the importance of the CRKP carriers identification, especially in the setting of high-risk haematologic patient.

The high colonization pressure favors CRKP diffusion [19–21]. The workload due to the concomitant hospitalization of a high number of CRKP carriers kept under contact precaution may increase the risk of isolation precaution transgressions [20, 21]. Moreover, in our experience, the occurrence of clinical emergencies in CRKP-positive patients seems to have further facilitated and accelerated the spread within the Unit: in the majority of patients we documented the CRKP acquisition after the occurrence of CRKP bacteremias or deaths in the Unit where they were hospitalized, and in 42% of cases within the following 3 days. Patient admissions or temporary transfer and readmissions from other wards where they could acquire CRKP may further increase colonization pressure [20, 21]. This might have happened in 27% of our cases, frustrating the efforts made to prevent CRKP spread and, in our opinion, also suggesting strict caution in the management of patients outside controlled wards.

Colonization was detected during neutropenia in half of the patients. We observed that the 26% of carriers had proved negative at the initial screening for CRKP carriage, and had the first CRKP positive swab collected after the onset of profound neutropenia and the initiation of fluoroquinololone prophylaxis, highlighting the role of timing and frequency of rectal controls. The rate (58%) of rectal carriers who developed bacteremia resulted much higher than the 39% reported in haematological patients in a multicentre prospective study [22], and the 26 and 40% reported in auto- and allo-stem cell transplant recipients, respectively [11]. This difference may be explained both by the high frequency of screenings - which prevented the possible underestimation of CRKP-carriers rate - and the high-risk level of our colonized population who were mainly acute leukemia patients (68%), AML in the 54% of cases. In our study, all the 9 AML patients who were CRKP carriers developed CRKP bacteremia, and the AML status was the only independent risk factor identified in CRKP carriers for bacteremia. We believe that the only strategy to reduce the incidence of CRKP bacteremias is to decrease the number of AML patients who are CRKP carriers, with active control measures against CRKP spread.

Overall, 45% of all patients harbouring CRKP and 37% of CRKP rectal carriers died of CRKP bacteremia. We found a higher mortality rate of CRKP bacteremia (71.4, 36% within 72–96 h) if compared to the 57.6% (8) and 53% (10) reported in haematological patients. This could be related, also in this circumstance, to the higher risk of our patients who developed bacteremia, of which 93% had acute leukemia, 86% AML, compared with 54% (8) and 57% (10) AML patients with CRKP bacteremia reported. All the patients who died for CRKP bacteremia had AML (they represented the 83% of AML patients who developed CRKP bacteremia) and all died for CRKP bacteremia during the first hospitalization as CRKP-positive patients (7 had become CRKP colonized and 3 developed the bacteremia without any previous microbiological evidence of CRKP colonization).

As already described in haematological patients [8, 10, 11], the majority (86%) of CRKP bacteremias occurred during neutropenia but, unlike other experiences [10], the 71.4% of bacteremias developed in patients already receiving an antibacterial therapy without in vitro activity against CRKP, and the 80% of these CRKP breakthrough bacteremias were fatal.

Even if CRKP bacteremias are usually preceded by rectal colonization [10, 11, 22], in our analysis previous CRKP colonization did not result as a risk factor for bacteremia, while AML did. In particular, we noted that in neutropenic patients colonization may rapidly progress in bacteremia, and bacteremia can develop before the detection of CRKP colonization or the availability of positive microbiological results. In our experience, despite the weekly CRKP rectal screening, 50% of all CRKP bacteremias and 70% of fatal CRKP bacteremias developed in patients not recognized as CRKP carriers, further reducing the possibility of an initial appropriate treatment. This may have contributed, together with the high rate of CRKP breakthrough bacteremias - of which 80% fatal - and with the initial lack of experience, to the delay in active drug administration and the high mortality rate of CRKP bacteremias.

The optimal treatment of infections caused by CRKP is still not defined [7, 23–25]. To improve survival in bacteremias a combined treatment with 2 or more in vitro active drugs (e.g.,polymixins, tigecycline, fosfomycin, gentamicin), including also carbapenems, is suggested [23–31], particularly as initial treatment [11, 23] and in high risk patients [9, 11, 24, 25]. To reduce the delay in adequate treatment of CRKP infection and the mortality rate, antibiotic combinations active against CRKP are suggested as empiric treatment of febrile neutropenia in CRKP colonized haematological patients [11]. In our experience, the initial adequate antibiotic therapy resulted the only condition able to protect against death and, like in other studies [9, 10], a very low rate of fatal CRKP bacteremias (20%) received active drugs as initial treatment. The emergence of antibiotic resistance is however a primary concern [26–31] and to avoid its fearsome increase, we believe that the extensive use of antibiotics active against CRKP for empiric treatments, or even more for the eradication of CRKP carriage [32–34] should be cautious and thoughtful also in CRKP endemicity, as in our country. The susceptibility profile of our CRKP isolates highlights the limited therapeutic options available and, in agreement with other reports [35], we observed the 86% of mortality in colistin-resistant CRKP bacteremia.

The low number of patients included in the study, the monocentric design and the lack of genotyping analysis of the CRKP isolates represent a limitation of the present analysis, however the study provides consistent data about the clinical impact of CRKP spread and CRKP infections in patients with haematological malignancies.

## Conclusions

The careful identification of carriers is confirmed critical to prevent the spread of CRKP also in haematological patients. Colonized AML patients resulted at high risk of CRKP bacteremia and poor outcome, and the initial therapy with active antibiotics may be effective to improve survival. Thus, in the setting of high CRKP pressure and high-risk haematological patients colonized by CRKP, particularly AML patients, the occurrence of a CRKP bacteremia should be strongly suspected in case of febrile neutropenia and timely empiric administration of combinations active against CRKP could be appropriate. We are currently conducting a prospective study to better define which haematological patients may benefit from this strategy.

## Abbreviation

AML: Acute myeloid leukemia
CRKP: Carbapenem-resistant *Klebsiella pneumoniae*
MDR: Multidrug-resistant
